# Supplementary material for: Spatially resolved rewiring of mitochondria-lipid droplet interactions in hepatic lipid homeostasis
Source: bioRxiv. 2024 Dec 12:2024.12.10.627730. Preprint. [Version 1] doi: 10.1101/2024.12.10.627730 (PMC11722523; doi:10.1101/2024.12.10.627730)
Supplement: Supplement 1 [file NIHPP2024.12.10.627730v1-supplement-1.pdf]

bioRxiv preprint doi: https://doi.org/10.1101/2024.12.10.627730; this version posted December 12, 2024. The copyright holder for this preprint (which was not certified by peer review) is the author/funder. This article is a US Government work. It is not subject to copyright under 17 USC 105 and is also made available for use under a CC0 license.

Supplementary Figures

Supplement Figure 1. Single-cell phenotyping of LD and mitochondrial topology in 3D. (A) Anatomical organization of the mouse liver lobule. The model was created with Biorender.com. **(B)** Representative projection of z-stack from mtDendra2 mouse visualizing mitochondria (green), lipid droplets (magenta), and actin (yellow). Scale bar 10 μm. **(C)** 3D surfaces of mitochondria (green), LDs (magenta), and actin (yellow) at a single-cell resolution. Cells are numbered from 1 to 12 on the PV-CV axis. Scale bar 2 μm **(D)** Quantification of mitochondrial sphericity and de nsity. **(E)** Quantification of lipid droplet volume and count. **(F)** Quantification of cell volume.

PV: portal vein; CV: central vein; LD: lipid droplet.

bioRxiv preprint doi: https://doi.org/10.1101/2024.12.10.627730; this version posted December 12, 2024. The copyright holder for this preprint (which was not certified by peer review) is the author/funder. This article is a US Government work. It is not subject to copyright under 17 USC 105 and is also made available for use under a CC0 license.

Supplement Figure 2. Lipid droplet accumulation in livers from mice fed WD for 4 weeks. (A) Confocal images of liver sections from C57BL6/J mice fed a control diet or a Western Diet as indicated. Lipids were labeled with LipidTox (white). **(B)** H&E staining images of liver sections from mice fed with control or western diet for 4 weeks. Scale bar 100 μm PP: periportal; PC: pericentral; WD: western diet.

Supplement Figure 3. Fasting induces Mitochondria-LD interactions in WD-fed mice. (A) Confocal images of liver sections from mtDendra2 (green) transgenic mice fed control, WD, overnight fasted or WD-overnight fasted. LDs were labeled with Lipidtox (magenta) and actin with phalloidin (yellow). Colocalization between mitochondria and LD is shown in white. Scale bar 3μm. **(B)** Quantification of mitochondria-LD colocalization under different dietary conditions. Statistical significance was calculated using two-tailed unpaired Student's *t*-test. Data presented as mean ± SD from $n = 5$ independent experiments. *$p < 0.05$, **$p < 0.01$, ***$p < 0.001$, ****$p < 0.0001$. WD: western diet; LD: lipid droplets.

Supplement Figure 4. Fatty acids drive ketogenesis, not OXPHOS, during fasting. **(A)** Venn diagram depicting PP or PC signature proteins in fed and fasted mice. **(B)** GO enrichment analysis for the proteomics data in the spatially sorted cell. **(C)** Heatmap showing levels of proteins in the mitochondrial respiratory complex in spatially sorted hepatocytes from fed and fasted mice. Values were derived from the means of three independent experiments. Basal and maximum respiration **(D-E)** and ATP production **(F)** in spatially sorted hepatocytes. Oxygen consumption rates (OCR) were measured with XF Mito Stress Test Kit, and Seahorse XF96 Analyzer. Samples were normalized to cell number. Statistical significance was calculated with two-way ANOVA. Data presented as mean ± SD from $n = 4$ independent experiments. **(G)** Liver beta-hydroxybutyrate concentrations from control fed or overnight fasted mice Data presented as mean ± SD from $n = 3$ independent experiments. Statistical significance was calculated using two-tailed unpaired Student's *t*-test.

bioRxiv preprint doi: https://doi.org/10.1101/2024.12.10.627730; this version posted December 12, 2024. The copyright holder for this preprint (which was not certified by peer review) is the author/funder. This article is a US Government work. It is not subject to copyright under 17 USC 105 and is also made available for use under a CC0 license.

Supplement Figure 5. *In vivo* overexpression of PLIN5.

(A) Relative Plin5 mRNA expression in mouse livers is presented as the mean ± SEM from n=5 except for WD mice expressing S155E and CΔ (1-424) where n=4. **(B)** Representative immunoblot and quantification of PLIN5 protein levels from one mouse per group. An antibody generated against the human PLIN5 carboxy terminus (upper panel) detects endogenous and exogenous full-length PLIN5 but does not detect the CΔ (1-424) variant. PLIN5 levels are relative to endogenous in the Null CNTR mouse. An antibody generated against amino acids within the amino terminus of human PLIN5 (lower panel) detects all forms of exogenous PLIN5 but does not detect endogenous levels in Null CNTR mice. PLIN5 levels are relative to WT exogenous levels in CNTR mice. Full-length PLIN5 migrates at 50 kDa and the CΔ (1-424) PLIN5 migrates slightly below 50 kDa. An intense non-specific >50kDa band is present in all samples and a <50kDa band of undetermined origin is seen in WD-fed mouse samples.

Supplement Figure 6. *In vivo* remodeling of mitochondria-LDs interactions. (A) Representative confocal images of liver sections from control (CNTR) diet-fed mice overexpressing PLIN5 variants. Images show mitochondria (green), LDs (magenta), and actin (yellow). PV: portal vein; CV: central vein. WT: wild type.

Supplement Figure 7. Overexpression of PLIN5 variants in WD-fed mice. (A) Representative confocal images of liver sections from Western Diet (WD)-fed mice overexpressing PLIN5 variants. Images show mitochondria (green), LDs (magenta), and actin (yellow). **(B)** Weight gain in mice overexpressing PLIN5 variants and fed CNTR or WD. **(C)** Serum fatty acid levels and **(D)** Serum cholesterol levels in mice overexpressing PLIN5 variants and fed CNTR or WD. Statistical significance was calculated with two-way ANOVA. Data presented as mean ± SD from $n = 5$ independent experiments. PV: portal vein; CV: central vein; WT: wild type. CNTR: control; WD: western diet.

bioRxiv preprint doi: https://doi.org/10.1101/2024.12.10.627730; this version posted December 12, 2024. The copyright holder for this preprint (which was not certified by peer review) is the author/funder. This article is a US Government work. It is not subject to copyright under 17 USC 105 and is also made available for use under a CC0 license.

Supplement table 1:

Antibodies	Source	Identifier	Dilution
APC anti-mouse CD73 antibody	Biolegend	127210	1:150
Alexa Fluor® 594 anti-mouse/human CD324 (E-Cadherin) Antibody	Biolegend	147306	1:100
Anti-mouse IgG, HRP-linked Antibody	Cell Signaling	3662S	1:10000
Anti-rabbit IgG, HRP-linked antibody	Cell Signaling	7076S	1:10000
Cyclophilin B (D1V5J) Rabbit monoclonal antibody	Cell Signaling	43603S	1:1000

bioRxiv preprint doi: https://doi.org/10.1101/2024.12.10.627730; this version posted December 12, 2024. The copyright holder for this preprint (which was not certified by peer review) is the author/funder. This article is a US Government work. It is not subject to copyright under 17 USC 105 and is also made available for use under a CC0 license.

β-actin (8H10D10) Mouse monoclonal antibody	Cell Signaling	3700S	1:1000
β-tubulin (9F3) Rabbit monoclonal antibody	Cell Signaling	2128S	1:1000
Goat anti-Rabbit IgG (H+L) Cross-Absorbed Secondary Antibody, Alexa Fluor 568	Invitrogen™	A11011	1:400
Alexa Fluor™ 568 Phalloidin	Invitrogen™	A12380	1:100
Alexa Fluor™ 647 Phalloidin	Invitrogen™	A22287	1:100
Perilipin 5 Polyclonal antibody	Proteintech	26051-1-AP	1:2000
OXPAT Polyclonal Antibody	Invitrogen™	PA5-114352	1:500
GAPDH	Cell Signaling	2118S	1:1000

**Dyes**		
Alexa Fluor™ 568 Phalloidin	Thermo Fisher	A12380
HSC LipidTOX™ Deep Red Neutral Lipid Stain, for cellular imaging	Thermo Fisher	H34477
**Buffers**		
RIPA Lysis buffer	Thermo Fisher	J62524-AE

bioRxiv preprint doi: https://doi.org/10.1101/2024.12.10.627730; this version posted December 12, 2024. The copyright holder for this preprint (which was not certified by peer review) is the author/funder. This article is a US Government work. It is not subject to copyright under 17 USC 105 and is also made available for use under a CC0 license.

Thermo Scientific™ Halt™ Protease and Phosphatase Inhibitor Cocktail (100X)	Thermo Fisher	78442
Prec Plus Protein Dual Color Standards	Bio-Rad	1610374
10X Tris/Glycine/SDS	Bio-Rad	1610732
2X Laemmli Sample Buffer	Bio-Rad	1610737
Clarity Western ECL Substrate	Bio-Rad	1705061
Seahorse XF base medium, without phenol red, 500 mL	Aglient	103335-100
Seahorse XF 1.0 M glucose solution, 50 mL	Aglient	103577-100
Seahorse XF 100 mM pyruvate solution, 50 mL	Aglient	103578-100
Seahorse XF 200 mM glutamine solution, 50 mL	Aglient	103579-100
Seahorse XF Calibrant Solution 500 mL	Aglient	100840-000
Seahorse XF Cell Mito Stress Test kit	Aglient	103010-100
Seahorse XF Mito Fuel Flex Test kit	Aglient	103260-100
Collage I, Rat Tail, 100 mg	Corning	354236
Phosphate Buffered Saline, pH 7.2, 1X	Quality Biological	111-056-101
Hanks' Balanced Saline pH 7.2, 1X	Thermo Fisher	14174-095
Acetic acid	Sigma Aldrich	A6283

bioRxiv preprint doi: https://doi.org/10.1101/2024.12.10.627730; this version posted December 12, 2024. The copyright holder for this preprint (which was not certified by peer review) is the author/funder. This article is a US Government work. It is not subject to copyright under 17 USC 105 and is also made available for use under a CC0 license.

Collagenase from Clostridium histolyticum	Sigma Aldrich	C5138
Krebs-Henseleit Buffer Modified	Sigma Aldrich	K3753
Calcium chloride solution	Sigma Aldrich	21115
Sodium bicarbonate	Sigma Aldrich	S6014
Ethylenediaminetetraacetic acid disodium salt solution	Sigma Aldrich	03690
HEPES solution	Sigma Aldrich	H0887
Fetal Bovine Serum	Thermo Fisher	26140079
TMTpro™ 16plex Label Reagent set	Thermo Fisher	A44520
HiSelect ™ Phosphopeptide enrichment kit	Thermo Fisher	A32992
High Select TM Phosphopeptide Enrichment kit	Thermo Fisher	A32993
Trypsin Platinum	Promega	VA9000
Urea	Sigma Aldrich	51457
Methanol	Sigma Aldrich	34860
Trifluoroacetic acid	Sigma Aldrich	302031
Acetonitrile solution	Sigma Aldrich	900686
**qPCR**		
Plin5	Thermo Fisher	Mm00508854_m1
Tbp	Thermo Fisher	Mm01277041_m1
Taqman Fast Advanced Master Mix	Thermo Fisher	4444963

bioRxiv preprint doi: https://doi.org/10.1101/2024.12.10.627730; this version posted December 12, 2024. The copyright holder for this preprint (which was not certified by peer review) is the author/funder. This article is a US Government work. It is not subject to copyright under 17 USC 105 and is also made available for use under a CC0 license.

High-Capacity RNA to cDNA kit	Thermo Fisher	4388950
RNeasy mini kit	Qiagen	74104
**Assay**		
Pierce™ BCA Protein Assay kit	Thermo Fisher	23225
Triglyceride (TG) Colorimetric assay kit	Elabscience	E-BC-K261-M
**Other**		
Pierce™ Peptide Desalting Spin Columns	Thermo Fisher	89851
Seahorse XF96 V3 PS culture Microplates	Aglient	101085-004
Mini-Protein® TGX™ Precast Gels	Bio-Rad	4568084
Trans-Blot Turbo NC Transfer Packs	Bio-Rad	1704158

bioRxiv preprint doi: https://doi.org/10.1101/2024.12.10.627730; this version posted December 12, 2024. The copyright holder for this preprint (which was not certified by peer review) is the author/funder. This article is a US Government work. It is not subject to copyright under 17 USC 105 and is also made available for use under a CC0 license.

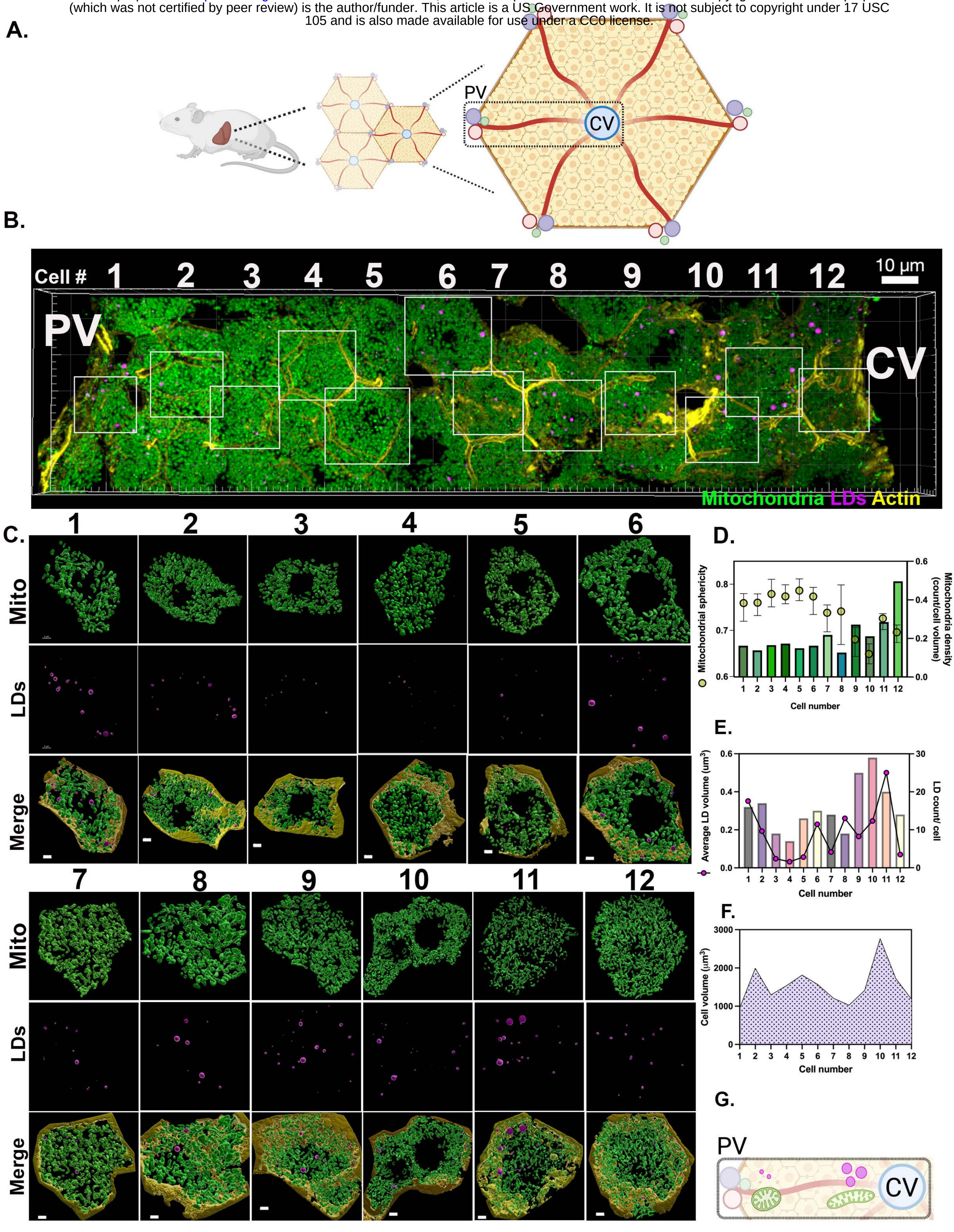

Figure S1. Single cell phenotyping of LD and mitochondria topology in 3D (scale 2microns)

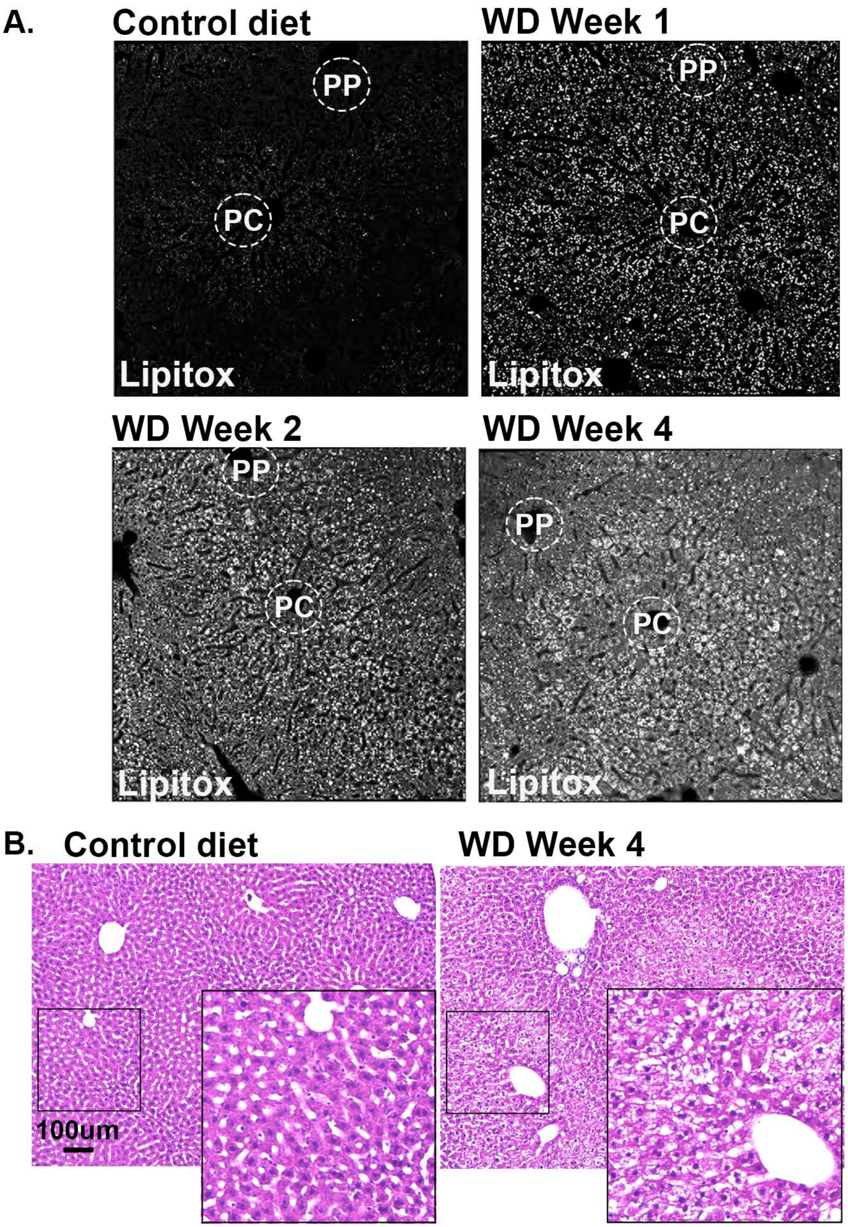

Figure S2. Lipid droplets buildup in mice fed WD for 4 weeks

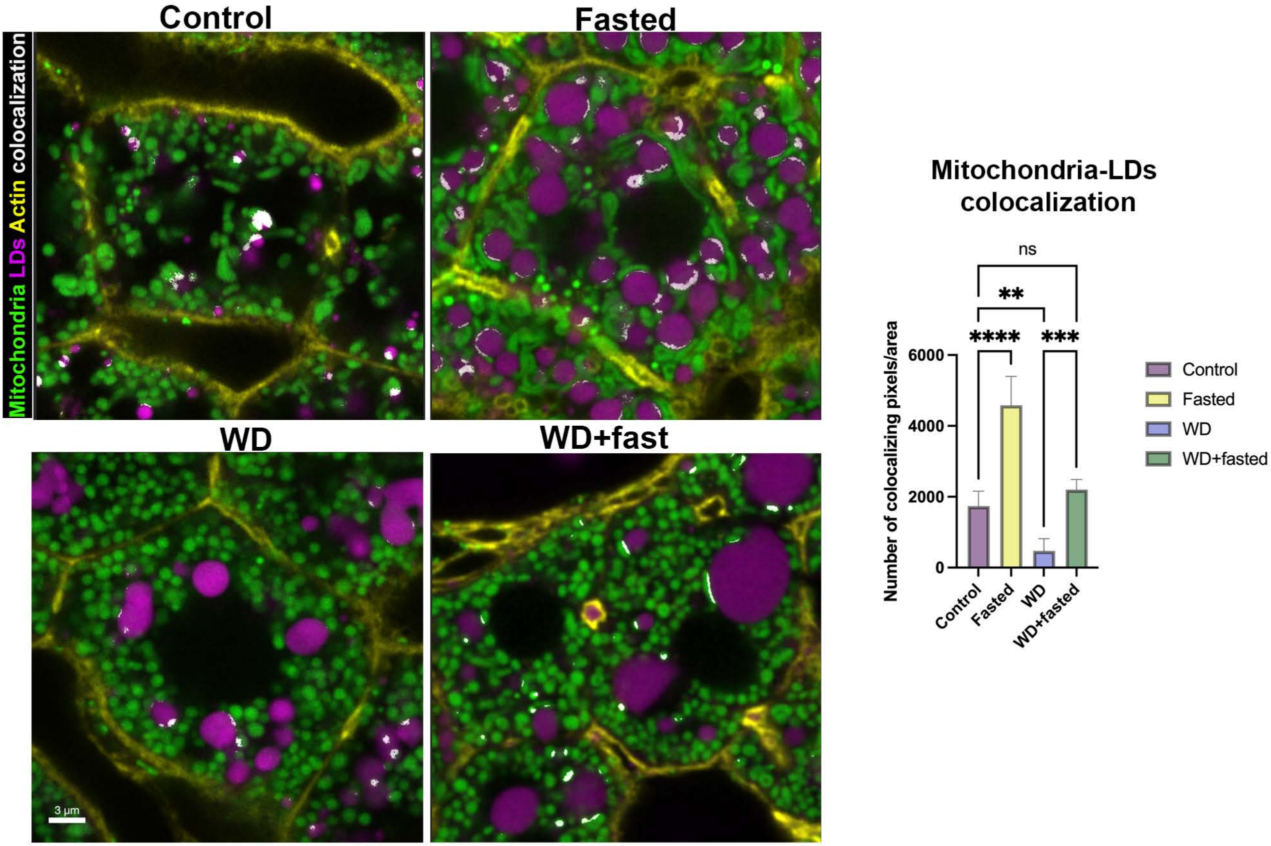

Fig. S3 Fasting induces Mitochondria-LD interactions in WD-fed mice

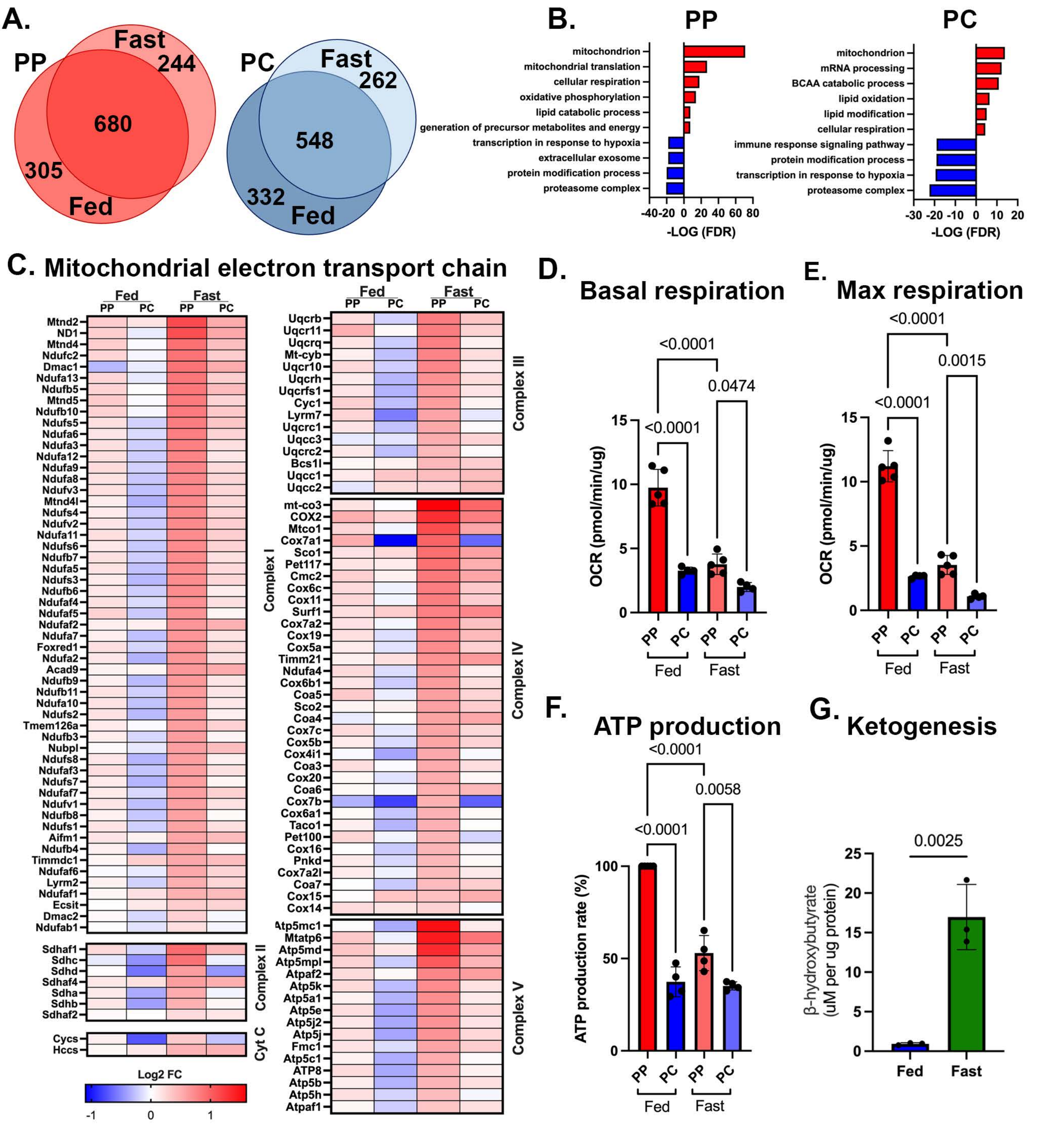

Fig. S4 Fatty acids drive ketogenesis, not OXPHOS during fasting

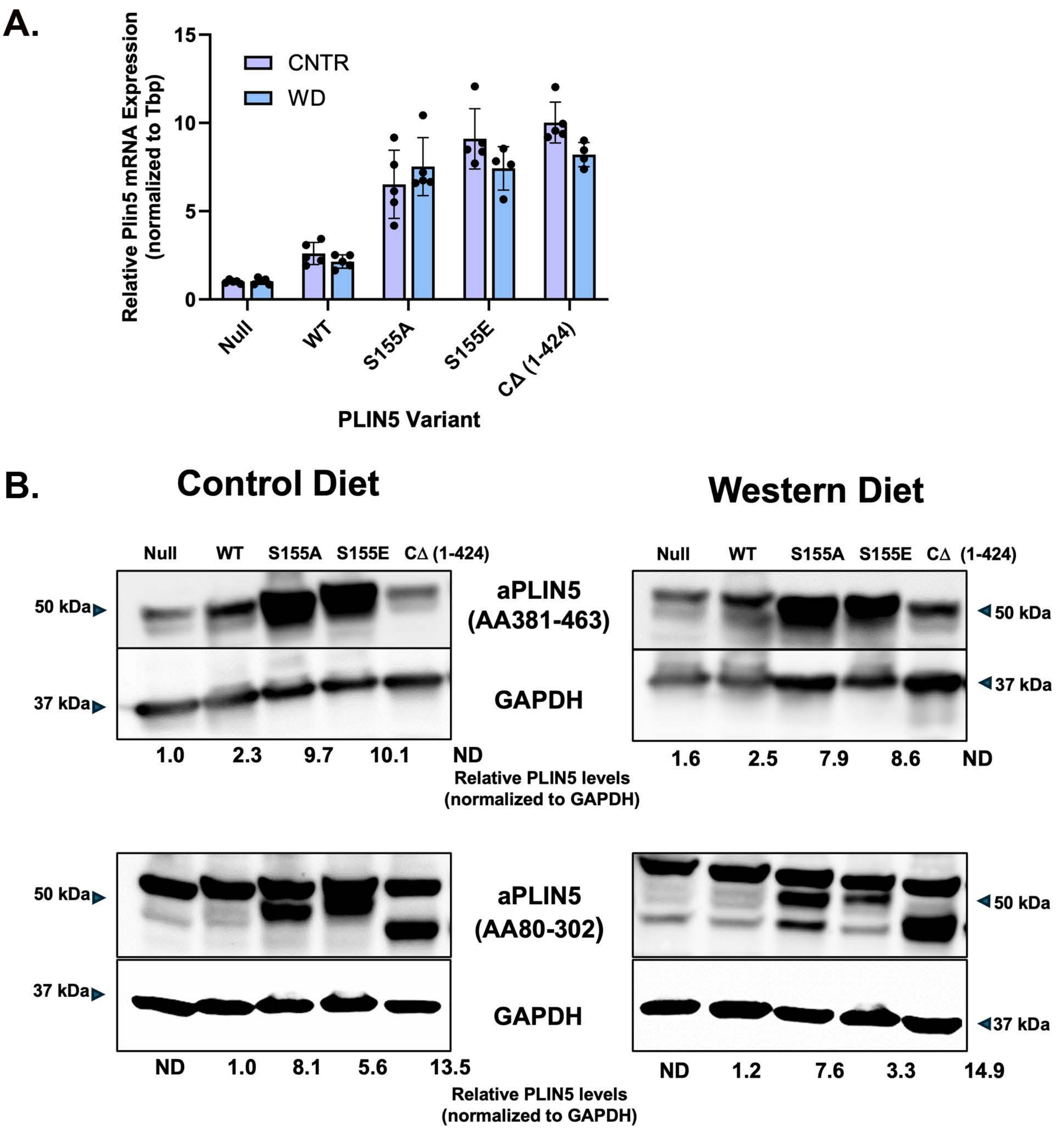

Fig. S5 Overexpression of PLIN5

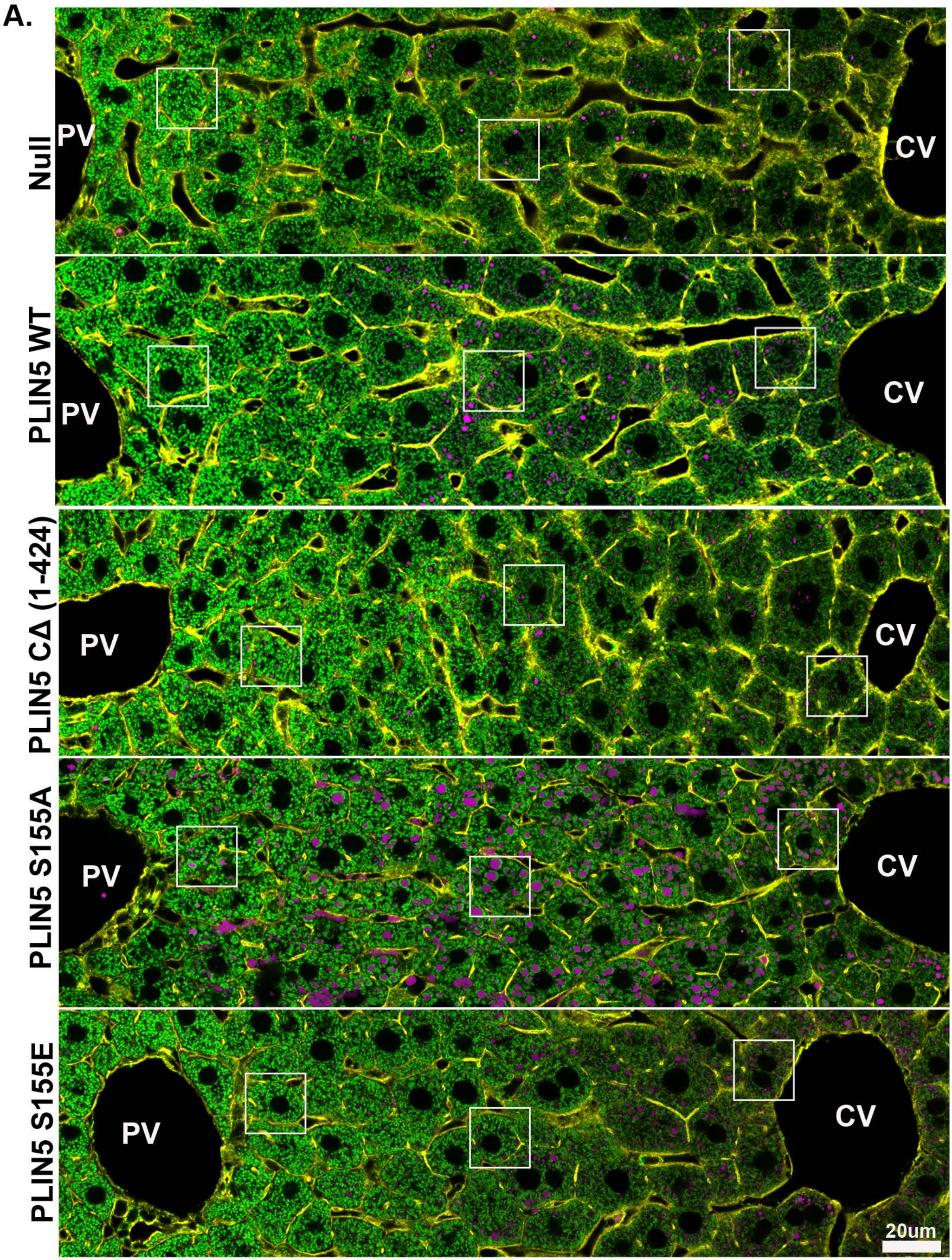

Fig. S6 In vivo remodeling of mitochondria-LDs interactions

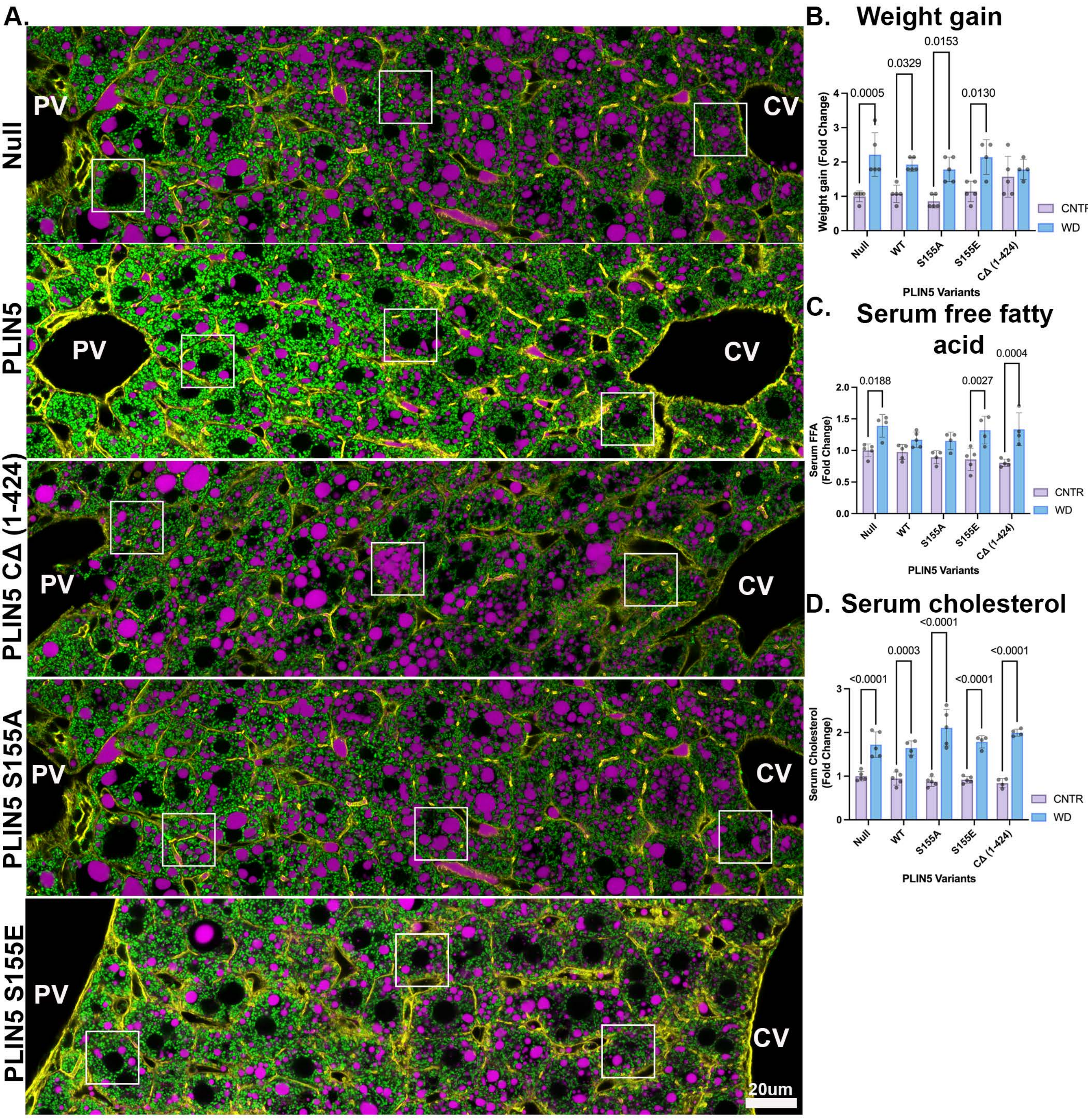

Fig. S7 Over expression of PLIN5 variants in WD-fed mice
